# Supplementary material for: In Vitro Anti-NTHi Activity of Haemophilin-Producing Strains of Haemophilus haemolyticus
Source: Pathogens. 2020 Mar 25;9(4):243. doi: 10.3390/pathogens9040243 (PMC7238096; doi:10.3390/pathogens9040243)
Supplement: Supplementary file 1 [file pathogens-09-00243-s001.pdf]

pathogens

MDPI

Article

In Vitro Anti-NTHi Activity of Haemophilin-Producing Strains of *Haemophilus haemolyticus*

Brianna Atto [1,*], Roger Latham [2], Dale Kunde [1], David A Gell [2] and Stephen Tristram [1,*]

[1] School of Health Sciences, University of Tasmania, Newnham Drive, Launceston, TAS, 7248, Australia; dale.kunde@utas.edu.au
[2] School of Medicine, University of Tasmania, 17 Liverpool Street, Hobart, TAS, 7000, Australia; roger.latham@utas.edu.au (R.L.), david.gell@utas.edu.au (D.A.G.)
* Correspondence: brianna.atto@utas.edu.au (B.A.), stephen.tristram@utas.edu.au (S.T.)

Received: 27 February 2020; Accepted: 22 March 2020; Published: date

Supplementary material

S1. Methods

S1.1. Triplex Real-Time PCR Assay Design, Optimisation and Validation

A triplex PCR assay was designed to detect and quantify NTHi, Hh and the *HPL* ORF. The targets used for discrimination of Hh (*HypD*) and NTHi (*SiaT*) have previously been described and validated [1]. For detection of the *HPL* ORF, primers were designed based on the *HPL* ORF of Hh-BW1 (GenBank MN720274) [2]. Primer specificity was confirmed using discontiguous megaBLAST analysis, performed across 115 complete and 862 draft genome assemblies for *Haemophilus.spp,* available from Genbank. A nonredundant nucleotide (nr/nt) collection BLAST search was also conducted to determine amplicon specificity in non-*Haemophilus* genomes.

Following optimisation in singleplex format, the three assays were merged into triplex format. Annealing temperature was optimised using an 8-step temperature gradient ranging from 53–63 °C. Specificity of amplicons was determined by gel electrophoresis, and the optimal temperature was selected based on highest yield of amplicons of the correct size in the absence of nonspecific amplification. PCR specificity for NTHi, Hh and *HPL* was determined using 13 Hh strains with varying *HPL* sequence similarity to BW1, as previously defined [2], 13 Hi strains and 9 other genera representing common upper respiratory tract flora (see Supplementary Table 2).

Reaction efficiency of triplex reaction was determined using 10-fold dilutions of control strains over the range of 2 to 2×10^{-8} ng (Hi ATCC49247, Hh ATCC 33390 and Hh BW1). Limits of quantification (LoQ) values were determined for *hypD* and *siaT* targets in triplex format based on criteria where replicates at a given dilution with a cycles to threshold (Ct) standard deviation (σ) of $\geq$ 0.8 were considered to exceed the LoQ, with one or more amplification failures also deemed an LoQ failure. The upper LoQ value was not determined due to the unlikelihood of encountering such high-pathogen DNA concentrations in clinical specimens. The lower limit of detection was also determined for *HPL* target and defined from linearity data (Figure S1).

S1.2. Boiled DNA Extraction Protocol

Bacterial suspensions were heated to 99 °C for 10 min in a heat block with shaking (1000 rpm). The tube was vented and vortexed for 10 s, followed by a further 10 min incubation at 99 °C. Boiled suspensions were spun for 10,000 g for 10 min in a benchtop microcentrifuge. The supernatant was diluted 1/5 in molecular grade water for use in PCR reactions. Given the instability of gDNA extracted by this method, PCR was conducted immediately or stored at 4 °C for no more than 2 h before use.

S1.3. Validation of Boiled DNA Extraction Method

A series of 10-fold dilutions (10^0–10^{-10}) were made made from a heavy suspension (OD_{600} = 3.0) of either Hh-BW1 or NTHi ATCC 49427 in TSB. To detect the presence of PCR inhibititors, each dilution was tested in duplicate and a nonlinear regression was generated. A slope between −3.1 and −4.1 (equivalent to amplification efficiency of 75%–110%) and a correlation coefficient > 0.98 was considered acceptable [3].

To test the methods accuracy in quantifying both targets simultaneously, each combination of NTHi and Hh dilutions in the range of 10^0–10^{-6} was mixed (50:50) prior to extraction and subsequent PCR. This produced varying proportions of NTHi and Hh to mimic possible scenarios of co-culture.

To compare OD_{600} readings and GE, a growth curve experiment was conducted where 110 uL aliquots were taken from a 5 mL culture in TSB every hour for 8 h and at 18 and 28 h. The OD_{600} of each aliquot was measured in a 96-well microtiter plate (Infinite 200 PRO, Tecan Life Sciences). Of this aliquot, 10 uL was used to produce dilutions for quantitation by colony count on chocolate agar and 50 uL was taken for gDNA extraction by boiling.

S2. Results

S2.1. Validation of Triplex Real-Time PCR

In silico specificity for the *HPL* amplicon revealed 97%–100% primer and probe nucleotide sequence identity for 26 of 61 complete or draft Hh genomes available in Genbank. Sequence similarity to the *HPL* amplicon was detected in 20 *H. influenzae* genomes out of 757 complete and draft genome assemblies. However, all alignments contained a minimum of four SNPs in the reverse primer and Taqman probe and did not contain any sequence homology with the forward primer. *HPL* was also detected in three genome assemblies available for *Haemopilus quentini*, which was expected based on previous analysis of the *HPL* ORF [2]. Despite close relatedness to Hh, isolation of this strain has only been described in the genitourinary tract so is unlikely to be co-isolated from respiratory specimens [4]. Comparison of 11 previously sequenced HPL ORFs [2] to these databases yielded the same results, indicating high sensitivity to known HPL sequence variants (ranging from 85%–100% homology to BW1-HPL). PCR of these isolates, and additional Hh, Hi and common upper respiratory tract colonisers confirmed specificity for each target.

S2.2. Quantification by PCR of Boiled DNA is Comparable to Optical Density

Serial dilutions did not reveal the presence of PCR inhibitors as regression parameters produced amplification efficiency of 99.37% and 91.57% for the *SiaT* and *HypD* targets, respectively (Figure S2). Similarly, amplification efficiencies did not differ between species, and their measurement was not affected when present in dramatically different, or similar, ratios (Figure S3). Growth patterns generated by OD_{600} could be replicated by PCR using DNA extracted by boiling. This was observed visually and by regression statistics directly comparing OD_{600} to GE (Figure S4).

S3. Figures and Tables

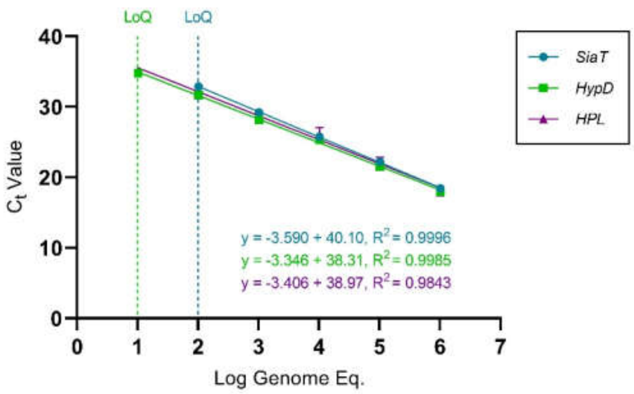

Figure S1. PCR efficiency and measure of LoQ and LoD. Shows PCR C_t values for the *SiaT*, *HypD* and *HPL* targets measured from serial dilutions of Hi, Hh^{HPL-} and Hh^{HPL+} control strain DNA over the range of 2 to 2 × 10^{-8} ng. Data points are represented as mean +/- SEM of two separate experiments, each conducted in duplicate.

Table S1. Bacteria strains used for triplex PCR specificity testing. In the case of Hh nucleotide sequence similarity to the *HPL* ORF of Hh-BW1 (model Hh-HPL^+) is shown.

Strains	Species	Similarity to BW1-*HPL* (%)
**BW1**	*H.haemolyticus*	100
**RHH122**	*H.haemolyticus*	100
**BW15**	*H.haemolyticus*	100
**BW18**	*H.haemolyticus*	100
**NF4**	*H.haemolyticus*	100
**NF5**	*H.haemolyticus*	100
**BW5**	*H.haemolyticus*	99
**CF14**	*H.haemolyticus*	96
**L19**	*H.haemolyticus*	85
**NF6**	*H.haemolyticus*	96
**NF11**	*H.haemolyticus*	96
**ATCC 33390**	*H.haemolyticus*	0
**NF1**	*H.haemolyticus*	0
**UTAS252**	*H. influenzae*	-
**L15**	*H. influenzae*	-
**CF31**	*H. influenzae*	-
**CF34**	*H. influenzae*	-
**CF48**	*H. influenzae*	-
**Ci2**	*H. influenzae*	-
**Ci3**	*H. influenzae*	-
**Ci5**	*H. influenzae*	-
**ATCC 10211**	*H. influenzae*	-
**ATCC 43163**	*H. influenzae*	-
**ATCC 49247**	*H. influenzae*	-
**NCTC 11315**	*H. influenzae*	-

**NTCT 4560**	*H. influenzae*	-
**NCTC 8618**	*S.salivarius*	-
**UTAS 8**	*S.pyogenes*	-
**UTAS14**	*S. pneumoniae*	-
**UTAS 203**	*Neisseria sp.*	-
**UTAS 387**	*S.aureus*	-
**ATCC 14990**	*S. epidermidis*	-
**ATCC 60193**	*C. albicans*	-
**ATCC 7901**	*H. parainfluenzae*	-
**UTAS 412**	*H.parahaemolyticus*	-

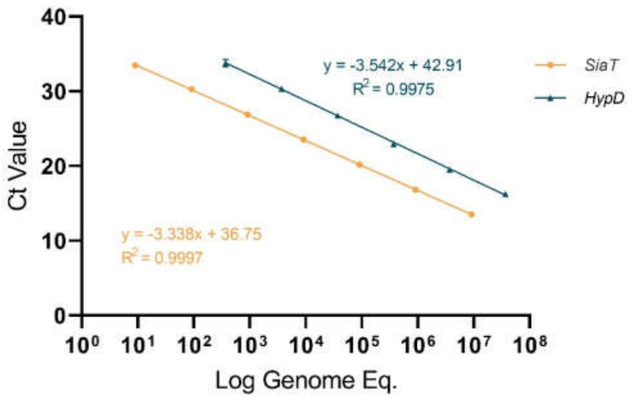

Figure S2. Detection of PCR inhibitors. Shows PCR C_t values for the *SiaT* and *HypD* targets measured from serial dilutions of DNA extracted by boiling. Data points represented as mean +/- SEM of two separate experiments, each conducted in quadruplicate.

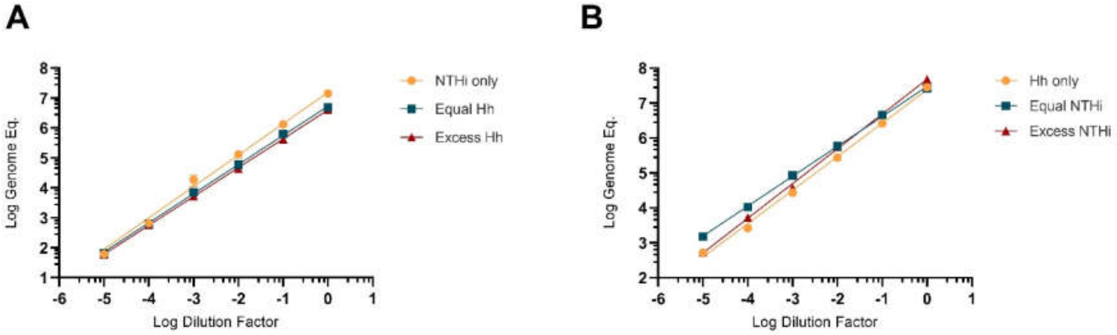

Figure S3. Extraction efficiency. Shows calculated genome equivalents from qrtPCR for (**A**) NTHi and (**B**) Hh measured from serial dilutions either alone or spiked with equal density, or excess density of Hh or NTHi, respectively. Data points represented as mean +/- SEM of two technical replicates. Nonlinear regression model determined there is no difference between slopes in any case.

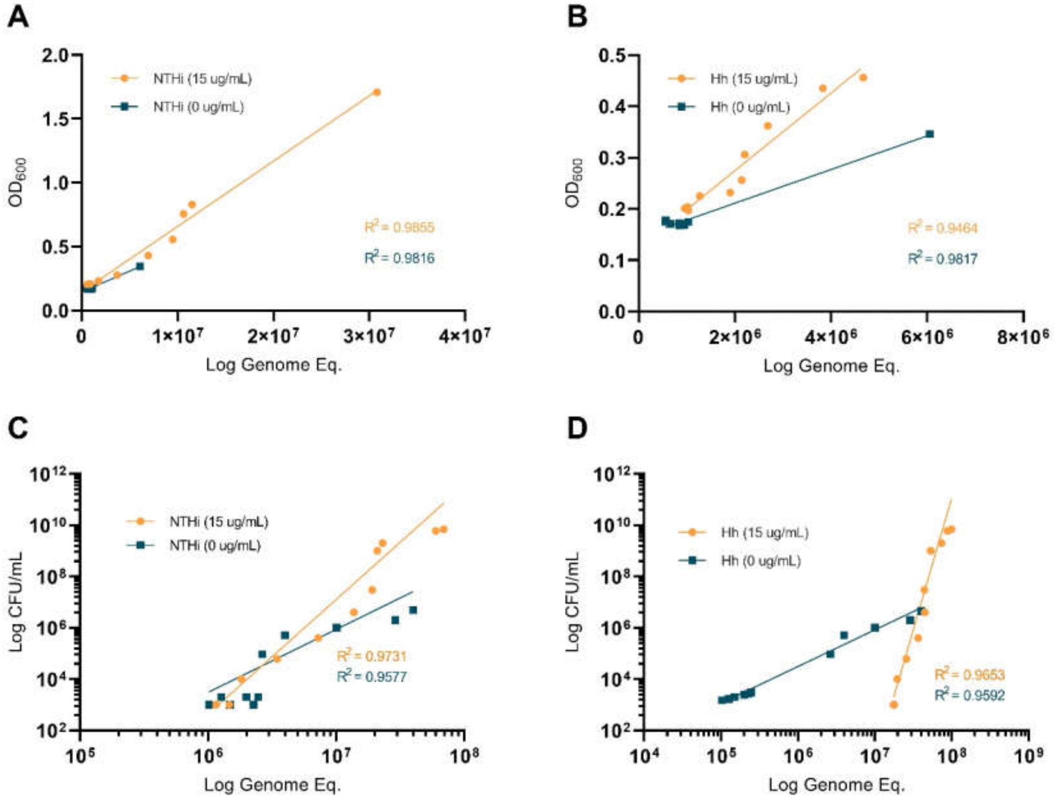

Figure S4. Comparison of genome equivalents and OD_{600} and colony counts (CFU/mL). Growth of NTHi and Hh in TSB over 16 h under heme-replete (15 ug/mL) or heme-starved (0 ug/mL) conditions as measured by OD_{600} in 96-well plates, or by calculation of GE from PCR of boiled DNA. Linear regression models show good correlation between quantification of NTHi, and Hh by OD_{600} (**A**, **B**) and colony counts (**C**, **D**). Data points represented as mean +/- SEM of duplicate extractions, measured by PCR in duplicate, over two separate experiments.

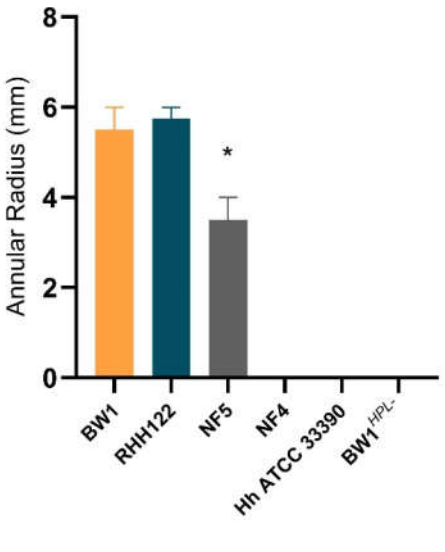

Figure S5. Baseline NTHi-inhibitory activity of HPL PCR positive Hh strains. Shows zones of inhibition, measured as annular radius (mm) of indicator strains (NTHi ATCC 49427) produced by ammonium precipitate extracts from Hh strains containing the HPL ORF. Mock extractions were

performed on Hh ATCC 33390 and BW1^{HPL-} as negative controls. Data points represented as mean +/- SEM of zones produced across the two indicators strains, from duplicate extractions.

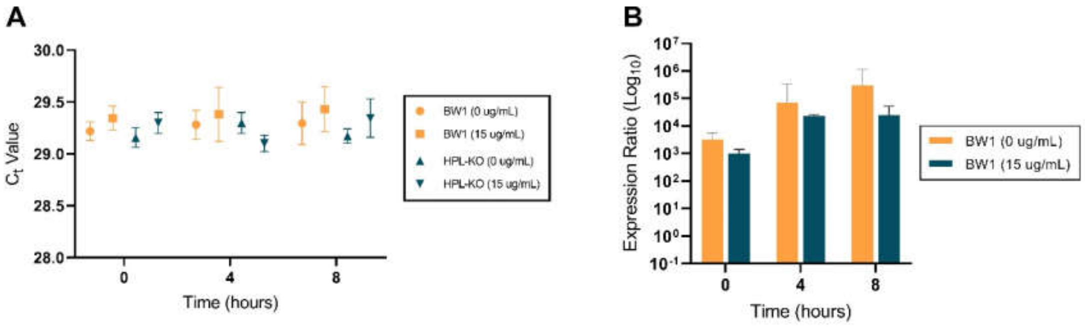

Figure S6. Validation of *HypD* (housekeeper gene) and *HPL.* (**A**) Expression of *HypD* by Hh-BW1 and HPL-KO during growth under heme-replete and heme-starved conditions. (**B**) Hh-BW1 expression of *HPL* during growth under heme-replete and heme-starved conditions. Data points represented as mean +/- SEM of two separate experiments, each with duplicate RNA extractions.

References

1. Price EP, Harris TM, Spargo J, Nosworthy E, Beissbarth J, Chang AB, et al. Simultaneous identification of Haemophilus influenzae and Haemophilus haemolyticus using real-time PCR. *Future Microbiol.* **2017**, *12(7)*, 585–593.
2. Latham RD, Torrado M, Atto B, Walshe JL, Wilson R, Guss JM, et al. A heme-binding protein produced by Haemophilus haemolyticus inhibits non-typeable Haemophilus influenzae. *Mol. Microbiol.* **2019**, doi: 10.1111/mmi.14426.
3. Waiblinger H-U, Grohmann L. Guidelines for validation of DNA extraction methods applied in subsequent PCR analysis of food and feed products for the presence of genetically modified material. *J. für Verbrauch. und Lebensm.* **2014**, *9*, 183–190.
4. Glover WA, Suarez CJ, Clarridge III J. Genotypic and phenotypic characterization and clinical significance of 'Haemophilus quentini'isolated from the urinary tract of adult men. *J. Med Microbiol.* **2011**, *60*,1689–1692.

© 2020 by the authors. Submitted for possible open access publication under the terms and conditions of the Creative Commons Attribution (CC BY) license (http://creativecommons.org/licenses/by/4.0/).
